# The role of R-spondin proteins in cancer biology

**DOI:** 10.1038/s41388-021-02059-y

**Published:** 2021-10-18

**Authors:** Eline J. ter Steege, Elvira R. M. Bakker

**Affiliations:** Department of Pathology, University Medical Center Utrecht, Utrecht, The Netherlands

**Keywords:** Oncogenes, Mechanisms of disease, Cancer stem cells, Predictive markers

## Abstract

R-spondin (RSPO) proteins constitute a family of four secreted glycoproteins (RSPO1–4) that have appeared as multipotent signaling ligands. The best-known molecular function of RSPOs lie within their capacity to agonize the Wnt/β-catenin signaling pathway. As RSPOs act upon cognate receptors LGR4/5/6 that are typically expressed by stem cells and progenitor cells, RSPO proteins importantly potentiate Wnt/β-catenin signaling especially within these proliferative stem cell compartments. Since multiple organs express LGR4/5/6 receptors and RSPO ligands within their stem cell niches, RSPOs can exert an influential role in stem cell regulation throughout the body. Inherently, over the last decade a multitude of reports implicated the deregulation of RSPOs in cancer development. First, *RSPO2* and *RSPO3* gene fusions with concomitant enhanced expression have been identified in colon cancer patients, and proposed as an alternative driver of Wnt/β-catenin hyperactivation that earmarks cancer in the colorectal tract. Moreover, the causal oncogenic capacity of RSPO3 overactivation has been demonstrated in the mouse intestine. As a paradigm organ in this field, most of current knowledge about RSPOs in cancer is derived from studies in the intestinal tract. However, *RSPO* gene fusions as well as enhanced *RSPO* expression have been reported in multiple additional cancer types, affecting different organs that involve divergent stem cell hierarchies. Importantly, the emerging oncogenic role of RSPO and its potential clinical utility as a therapeutic target have been recognized and investigated in preclinical and clinical settings. This review provides a survey of current knowledge on the role of RSPOs in cancer biology, addressing the different organs implicated, and of efforts made to explore intervention opportunities in cancer cases with RSPO overrepresentation, including the potential utilization of RSPO as novel therapeutic target itself.

## Introduction

The R-spondin (RSPO) family is represented by four genes *RSPO1, 2, 3*, and *4*, encoding like-named secreted signaling proteins. Homologs of RSPOs are present amongst vertebrates and typically contain a thrombospondin type I repeat (TSR) domain, explaining their historical names as *Human Protein With ThromboSpondin type I Repeat (hPWTSR)* and *Cysteine-rich single thrombospondin type I repeat containing protein* (*Cristin)* [1, 2]*. RSPO3* was the first member to be identified in a human fetal brain cDNA library in 2002, followed by the identification of mouse *Rspo1* in 2004 (ref. [1, 3]). As *Rspo1* expression was observed in the roof plate of the neural tube during mouse development, it was named Roof plate specific–Spondin (R-spondin). Subsequently, *RSPO2* and *RSPO4* were identified [4, 5]. Genetic mouse and human studies have revealed divergent and pivotal roles for the four RSPO members during development. Mutations in *RSPO1* are linked with female-to-male XX sex reversal and *Rspo1* knockout in mice revealed an important role in ovarian development [6, 7]. *Rspo2* is involved in limb and respiratory tract development as well as craniofacial patterning and morphogenesis [8–11]. *Rspo3* is essential for angiogenesis, vasculogenesis and placental development whereas genetic mutations in *RSPO4* were detected in people with anonychia, characterized by the absence of finger and toe nails [12–17].

**Fig. 1:**
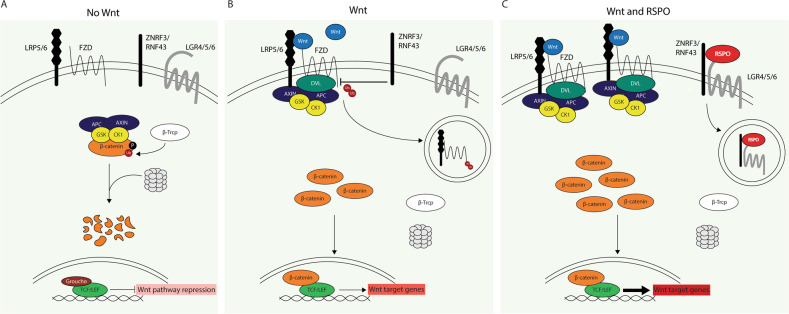
The canonical Wnt pathway and the potentiating effect of *RSPO*. **A** In the absence of canonical Wnt ligands the central destruction complex induces β-catenin degradation, restraining the transcription of Wnt target genes. **B** Canonical Wnt ligands induce dissociation of β-catenin from the degradation complex, leading to β-catenin accumulation, nuclear translocation and transcription of Wnt target genes. Ubiquitin ligases ZNRF3/RNF43 negatively regulate the Wnt pathway by internalizing and degrading membrane receptors LRP5/6 and FZD, thereby reducing Wnt receptor availability at the membrane. **C** RSPOs potentiate the canonical Wnt pathway by clearing negative regulators ZNRF3/RNF43 from the membrane, thereby increasing membranous Wnt receptor availability and potentiation of Wnt ligand-mediated pathway activation.

In 2004, *Xenopus* studies first described what is now the best-known molecular activity of R-spondin proteins: potentiation of the Wnt/β-catenin pathway, a crucial signaling pathway that regulates multiple fundamental processes including proliferation, stem cell control, tissue homeostasis and regeneration [5, 18]. Because of this fundamental role, the activity of the Wnt/β-catenin signaling pathway, in other words the downstream transcriptional activity of effector protein β-catenin, requires tight regulation which is executed at multiple levels. The central restraint of the pathway is provided by the intracellular APC containing destruction complex, which induces β-catenin degradation and as such inhibits the pathway (Fig. 1A). The pathway is activated upon binding of extracellular Wnt ligands to LRP5/6 and Frizzled (FZD) membrane receptors, leading to dissociation of β-catenin from the degradation complex, stabilization and nuclear translocation of β-catenin and subsequent transcriptional regulation of target genes in the nucleus (Fig. 1B). Another level of negative regulation is provided by ZNRF3 and RNF43, ubiquitin ligases that promote the degradation of LRP5/6 and FZD receptors, thereby reducing membranous Wnt receptor availability and subsequent downstream β-catenin signaling capacity [19, 20]. It is this latter ZNRF3/RNF43-mediated negative feedback loop that RSPO proteins interfere with, providing an additional level of canonical Wnt pathway regulation. All four RSPOs hold a conserved domain pattern composed of an N-terminal signal peptide, 2 cysteine rich furin like (FU1-FU2) domains, a thrombospondin (TSP) domain and a basic amino acid rich (BR) C-terminal domain. The FU1, FU2 and TSP domains enable RSPO proteins to bind ZNRF3/RNF43, Leucine-rich repeat-containing G-protein coupled receptors (LGR) 4–6 and heparin sulfate proteoglycans (HSPGs) respectively [2, 21–28]. Through interaction with ZNRF3/RNF43 and LGRs, RSPOs induce membrane clearance of ubiquitin ligases ZNRF3/RNF43, leading to enhanced Wnt receptor availability at the cell membrane and thereby potentiating Wnt ligand-mediated activation of the Wnt/β-catenin pathway (Fig. 1C)[19].

Despite the high homology among the four RSPOs, differences exist in their ability to bind LGRs and ZNRF3/RNF43 and to potentiate the Wnt/β-catenin pathway, where RSPO2 and RSPO3 show highest ZNRF3/RNF43 binding affinity and activity [21, 29]. Moreover, RSPO2 and RSPO3 can potentiate the Wnt/β-catenin pathway independently of LGR binding [27, 30]. This activity requires the binding of RSPOs to HSPGs with the TSP and BR domains in addition to binding to ZNRF3/RNF43 with the FU1 domain [27, 31]. Another study reported that RSPOs are also able to potentiate the Wnt/β-catenin pathway independent of ZNRF3/RNF43, where interaction of RSPO with LGR4 increases the affinity of scaffold protein IQGAP1 to bind DVL, resulting in LRP6 phosphorylation and potentiation of the Wnt/β-catenin pathway [32]. In addition to canonical Wnt signaling, RSPOs have also been implicated in non-canonical Wnt signaling in *Xenopus* embyros, where RSPO3 can modulate non-canonical Wnt/PCP signaling by binding to HSPG syndecan-4 and LGR4,5 to regulate gastrulation and head cartilage morphogenesis [28, 33]. Moreover, a recent *Xenopus* study described that RSPO3 exerts antagonistic effects on the BMP signaling route additionally, through binding of ZNRF3 and BMP receptor 1 A [34]. Thus, since their discovery multiple signaling activities have been attributed to RSPOs, especially in the regulation of canonical Wnt/β-catenin pathway but also beyond. In that perspective, more lessons will expectedly be learned considering the molecular activities of RSPOs.

The discovery that RSPOs represent ligands of the LGR4/5/6 receptors raised special interest, since these receptors are typically expressed by progenitor cells and stem cells, and as such the agonistic activity that RSPOs exert on Wnt/β-catenin signaling importantly influences the proliferative stem cell compartment [35–38]. As LGR5 was identified as a marker of stem cells in the intestine followed by the recognition of RSPOs representing LGR ligand, most knowledge on RSPO-LGR signaling currently exists in the field of the intestinal tract [33, 35, 39, 40]. However, RSPOs and LGR4/5/6 receptors are present in multiple organs and therefore RSPOs can influence stem cell regulation throughout the body. In accordance with this broad stem cell regulatory role, deregulated RSPO activity has increasingly been implicated in cancer development lately and *RSPO* alterations have been reported to occur in multiple cancer types as reviewed below (Table 1).

**Table 1:** *RSPO* alterations reported among cancer types.

Organ	RSPO alteration	References
Intestine	Gene fusions	
	*EIF3E-RSPO2*	[49–51]
	* PIEZO1-RSPO2*	[51]
	* NRIPI-RSPO2*	[52]
	* PRR15L-RSPO2*	[53]
	* PTPRK-RSPO3*	[49–52]
	Overexpression	
	* RSPO2*	[49, 50]
	* RSPO3*	[43, 49, 50]
Stomach	Gene fusions	
	* EMC2-RSPO2*	[67]
	* HNF4G-RSPO2*	[67]
	Overexpression	
	* RSPO3*	[64]
Breast	Overexpression	
	* RSPO2*	[113]
	* RSPO3*	[113, 115, 116]
	* RSPO4*	[113, 116]
Ovary	Overexpression	
	* RSPO1*	[116, 122]
	* RSPO3*	[116]
Prostate	Gene fusions	
	* GRHL2-RSPO2*	[131]
	Overexpression	
	* RSPO3*	[130]
	Downregulation	
	* RSPO3*	[132]
Liver	Gene fusions	
	* SINE-RSPO2*	[137]
	Overexpression	
	* RSPO2*	[136–138]
Lungs	Gene fusions	
	* EIF3E-RSPO2*	[141]
	* PTPRK-RSPO3*	[141]
	Overexpression	
	* RSPO3*	[116, 140]
Pancreas	Overexpression	
	* RSPO2*	[116, 142]
Bladder	Overexpression	
	* RSPO3*	[143]

### Gastrointestinal tract

#### Intestine

The intestinal epithelium displays an exceptionally rapid turnover that is controlled by tightly balanced molecular signaling in conjunction with unique cellular build-up of the crypt-villus architecture. Canonical Wnt/β-catenin signaling plays a central role in fueling proliferation and self-renewal in the crypt region, from where most progeny cells migrate towards the villus region whilst differentiating. The intestinal stem cell niche housed in the crypt region holds a refined composition of cycling stem cells, being protected and instructed by neighboring Paneth cells, quiescent stem cells and transient amplifying cells. Importantly, the cycling stem cells that fuel the continuous epithelial renewal express the RSPO receptor LGR5, whilst LGR4 is expressed more broadly on cycling stem cells, transient amplifying cells and Paneth cells [35, 41]. RSPO3 ligand is produced by stromal cells that lie in close proximity to the crypt stem cells both in mouse intestine and human colon [42–44]. Within this crypt environment, paracrine regulation of Wnt/β-catenin signaling by RSPO and Wnt ligands provide instructive signals to the intestinal stem cell niche, albeit in distinct manners [45]. Whereas Wnt ligands agonize canonical Wnt/β-catenin signaling, they are incapable of inducing renewal of LGR5^+^ stem cells on their own [45]. Instead, Wnt ligands induce RSPO receptor expression, thereby optimizing conditions for RSPO ligands to exert their effects. RSPO ligands in their turn induce the self-renewal and expansion of stem cells, as such dictating the size of the intestinal stem cell pool [45]. In case of intestinal injury, stromal RSPO3 expression is elevated and is demonstrated to be indispensable for epithelial regeneration by inducing Wnt/β-catenin signaling in differentiated cells, probably through the LGR4 receptor [46]. Of note, LGR5^+^ stem cells are dispensable in these epithelial regeneration processes, indicating that RSPO3 ligand is essential and dominantly instructive in epithelial repair in the gut [46–48]. Taken together, in the non-transformed intestine, RSPO3 is produced in the pericryptal stroma and plays a fundamental role in controlling stem cell numbers and epithelial recovery through activation of the Wnt/β-catenin pathway.

In line with the central role of Wnt/β-catenin signaling in intestinal stem cell maintenance, hyperactivation of this pathway is the hallmark feature of colorectal cancer (CRC). In the majority of CRC patients, this hyperactivation is caused by either inactivating *APC* mutations or activating mutations in the β-catenin gene *CTNNB1*, both resulting in constitutive activation of the Wnt/β-catenin pathway, independent of Wnt ligand binding. Importantly, in 2012 it was found that 4–10% of CRC patients harbor gene fusions of the *RSPO2* and *RSPO3* genes with *EIF3E* and *PTPRK* respectively, co-occurring with enhanced expression of the considerate *RSPO* gene [49, 50]. These *RSPO2* and *RSPO3* gene rearrangements were found mutually exclusive with other Wnt pathway mutations, though co-occurring with either *KRAS* or *BRAF* mutations, suggestively serving as an alternative mechanism to achieve hyperactivation of the Wnt/β-catenin pathway and to hold oncogenic capacity [49, 50]. Following up on the initial discovery of *RSPO* gene fusions in CRC patients, other studies identified additional gene fusions of *RSPO2* with *PIEZO1*, *NRIPI* and *PRR15L* and moreover, reported *RSPO* gene fusions to typically occur in traditional serrated adenoma (TSA) rather than conventional colon tumors [51–54]. In addition, another CRC patient subpopulation has been described that harbors high *RSPO3* expression levels but seem to lack *RSPO* gene fusions or alternative Wnt pathway mutations [43]. Instead, in these tumors the elevated levels of RSPO3 are produced by the stromal compartment, and in line, most of these cases were of the CMS4 mesenchymal subtype [43]. These data suggest that enhanced *RSPO3* expression by stromal cells can substitute for epithelial *RSPO* mutations in driving CRC. As RSPOs are secreted ligands, these findings support the plausibility that especially the cells that receive the RSPO signals, rather than the producing cells, determine the oncogenic response, therefore being most interesting in understanding the biology of RSPO-driven cancer. For CRC, the typical occurrence of *RSPO* gene fusions in TSA might be informative in this regard, and it has been proposed that this might point towards a different, TSA-like evolutionary trajectory for RSPO-mutant tumor development, distinct from conventional CRC [55]. However, details on the potential cell of origin and mutation selection along the tumorigenic cascade within RSPO-driven cancer remain to be unraveled.

Formal evidence for the causal oncogenic capacity of *Rspo3* was provided by a mouse study where conditional *Rspo3* overexpression consistently induced abundant intestinal tumor formation, demonstrating that augmentation of *Rspo3* levels is causative in driving tumorigenesis [56]. RSPO3-driven tumors showed major expansion of crypt cells including LGR5^+^ stem cells, quiescent stem cells, Paneth cells and LGR4^+^ cells with modestly increased Wnt/β-catenin signaling [56]. Thus, enhanced *Rspo3* levels induced a magnification of the proliferative, self-renewing crypt compartment. Adding up to the oncogenic capacity of *Rspo3* overexpression, another mouse study showed that also the transgenic expression of either *EIF3E-RSPO2* or *PTPRK-RSPO3* gene fusion causally drives the formation of intestinal tumors, which comparably show expansion of proliferative cells and ectopic Paneth cells [57]. Inversely, targeted anti-RSPO3 treatment in a *PTPRK-RSPO3* xenograft CRC model was shown to induce tumor differentiation whilst reducing growth, stem cell marker expression and canonical Wnt pathway activity [58]. Thus, these mouse studies demonstrated that RSPO gain of function, either through overexpression or genetic rearrangement, causally drives intestinal tumorigenesis, wherein deregulation of the proliferative stem cell compartment was shown to be involved. Notably, despite this and the occurrence of *EIF3E-RSPO2* fusions and enhanced *RSPO2* expression in CRC patients, some controversy exists considering the role of RSPO2 in CRC. Hence, RSPO2 has also been attributed tumor suppressive activities in CRC in some reports [59, 60].

In summary, during the last decade, studies in the intestinal tract have revealed that a subset of CRC patients harbors a gain in RSPO, which can act as oncogenic driver through fueling aberrant expansion of the crypt stem cell compartment. Currently, most of our knowledge on RSPOs in cancer is derived from studies in the intestine, and for this organ, aberrant RSPO activation is recognized as oncogenic driver.

#### Stomach

As in the intestine, Wnt signaling plays a crucial role in regulating epithelial turnover in the stomach and aberrant activation of the Wnt/β-catenin pathway is an established driver of gastric cancer [61–63]. In the homeostatic stomach, Wnt ligands and RSPO3 are expressed in the stroma neighboring the gland base that constitutes the gastric stem cell compartment [63, 64]. The stem cell compartment of mouse gastric antrum glands is composed of Lgr5^+^/Axin2^+^ cells at the base and more apical Lgr5^-^/Axin2^+^ cells [63]. Both these stem cell populations are capable of repopulating the gastric gland, giving rise to progenitor and differentiated cell types [37, 63–65]. The Lgr5^-^/Axin2^+^ cells appear to be the main driver of homeostatic epithelial turnover, repopulating the glands in 7 days, whereas Lgr5^+^/Axin2^+^ show relatively less proliferation and a gland turnover time of 10–14 days [63, 64]. In the stomach, RSPO3 is produced by myofibroblasts neighboring the stem cell compartment and plays a crucial role in regulating stem cell dynamics [64]. Interestingly, RSPO3 induces Lgr5^+^/Axin2^+^ stem cells to differentiate into secretory cells with antimicrobial activity, protecting the stem cell compartment against bacterial colonization [65]. In contrast, RSPO3 acts upon Lgr5^-^/Axin2^+^ cells by promoting their proliferation and expansion, probably through Lgr4 that is expressed on these cells [64]. Infection with *Helicobacter pylori (H. pylori)* enhances stromal *Rspo3* expression and leads to expansion of proliferative Axin2^+^ stem cells and hyperplasia [64]. Importantly, *H. pylori* infection represents the main risk factor for the development of gastric cancer. Enhanced proliferation of gastric stem cells driven by RSPO3 upon *H.pylori* infection might contribute to this increased risk for cancer development [63, 66]. Despite interesting links have been revealed among stem cell (de)regulation, RSPO3 and *H.pylori* infection in the stomach, more research is needed to further assess their possible interplay in gastric carcinogenesis. With regard to genetic alterations that might underlie RSPO deregulation in gastric cancer patients, current knowledge is relatively limited. Two cases of *RSPO2* gene fusions have been reported in gastric cancer patient-derived xenograft (PDX) material by one group [67].

### Steroid hormone regulated organs

#### Breast

The mammary gland represents another organ where both RSPO and Wnt/β-catenin signaling have been implicated in stem cell regulation during homeostasis and carcinogenesis [68–74]. Although at first glance this involvement might seem comparable to the benchmark situation in the intestinal tract, it is important to realize that the mammary gland is a totally different, uniquely organized epithelial structure that is primarily instructed by steroid hormones estrogen and progesterone. The bilayered mammary epithelium consists of outer basal cells and inner luminal cells, latter being further segregated into luminal progenitor cells and mature luminal cells that express the estrogen receptor (ER) and progesterone receptor (PR). Steroid hormones regulate the exceptionally dynamic remodeling events that occur during puberty, menstrual cycles, pregnancy, lactation and involution. These processes require tightly controlled self-renewal, and the mammary epithelium constitutes a complex and unique, yet incompletely clarified hierarchy of co-existing progenitor and stem cell populations [38, 71, 74–83]. Mammary stem cells (MaSC) with repopulating capacity were firstly described to be part of the basal population [84, 85]. More recent studies report stem cells both in basal and luminal populations, and Wnt/β-catenin signaling has been implicated in the regulation of MaSCs [68, 71, 72, 74, 78–83]. In human breast, RSPO3 is expressed in ALDH+ cells, a cell population that has been proposed to represent (cancer) stem cells and luminal progenitor cells [86–88]. In the mouse mammary gland, RSPO1 has emerged as a key regulator of MaSCs, leading to defects in side-branching and alveologenesis upon its depletion [70, 73, 89, 90]. RSPO1 is produced by luminal progenitor cells, in proximity to mature luminal cells that produce Wnt4, which together cooperate in promoting the self-renewal of MaSCs [70, 73]. Moreover, RSPO1 and Wnt4 are synchronously upregulated upon steroid hormone signals during pregnancy, leading to Wnt/β-catenin signaling potentiation and fueling the expansion of basal cells and luminal progenitor cells [70, 73]. This collaborative RSPO1-Wnt4 action seems to represent the actual downstream executor of stem cell regulation, in response to upstream steroid hormone signals.

In line with the extensive stem cell hierarchy in the mammary gland, breast cancer is exceptionally heterogeneous, and uniquely classified based on the expression of the hormone receptors ER, PR and human epidermal growth factor receptor 2 (HER2). Triple negative breast cancer (TNBC) that lacks expression of these three receptors is the most aggressive subtype with poorest prognosis and most limited options for targeted treatment. Activation of the canonical Wnt pathway in breast cancer has been reported regularly, amongst multiple subtypes, though an association has been proposed especially with TNBC [91–100]. In striking contrast to CRC however, the majority of breast tumors lack mutations in *APC* or *CTNNB1*, obscuring the mutational cause of reported intracellular Wnt activation [68, 93]. A possible explanation for this might lie in the different tissue-specific dosages of canonical Wnt signaling activation that support tumor growth, where tumor growth in the mammary epithelium favors a relatively weaker level of Wnt/β-catenin activation compared to its intestinal counterpart [101–103]. Also, activation of the Wnt pathway might result from alterations in other pathway members [68, 104–107]. In this regard, RSPOs might represent additional candidates, supported by the self-renewal promoting effects that RSPO exerts in the normal mammary gland. The first indications that RSPOs might potentially represent mammary oncogenes come from studies in which *Rspo1*, *Rspo2*, and *Rspo3* were identified as common integration sites of the mouse mammary tumor virus (MMTV) [108–111]. This was further supported by experiments where injection of cell lines overexpressing *Rspo2* or *Rspo3* in the mouse mammary gland resulted in mammary tumor formation, and distant metastases in case of *Rspo2* (ref. [110, 112]). With regard to RSPOs in breast cancer patients, some reports have suggested a protumorigenic role for overexpressed RSPOs, mostly based upon associative studies and in vitro data [113–115]. Overexpression of *RSPO2, RSPO3*, and *RSPO4* have been reported in breast tumors, with a particular occurrence in TNBC and being associated with reduced patient survival in case of *RSPO2* upregulation [113, 115, 116]. Notably, *EIF3E-RSPO2* fusion transcripts known to occur in CRC were not found in a group of 446 breast tumors tested [113]. This directed approach for these fusions specifically does however not exclude the possibility that other *RSPO* gene fusions might occur in breast cancer. Notably, the two cell lines HBcc-15 and BT549 that are derived from breast cancer patients do have *EIF3E-RSPO2* gene fusions, and siRNA-mediated inhibition of *RSPO2* in BT549 cells was shown to reduce the proliferation of this TNBC cell line [113]. Together these data point towards a protumorigenic role for RSPOs in breast cancer, though further research is needed to better establish this.

#### Ovary

In ovarian development RSPO1 has appeared as a crucial player, regulating female sex determination and ovarian differentiation in cooperation with Wnt4 (ref. [6, 7, 117–119]). RSPO1 and Wnt4 are expressed throughout ovarian development and influence cell proliferation and the entry of germ cells into meiosis by activating the Wnt/β-catenin signaling pathway [117, 119, 120]. In agreement with its essential role in ovarian development, the Wnt/β-catenin pathway has found to be frequently activated in ovarian cancer, being associated with epithelial-to-mesenchymal progression, chemotherapy resistance and poor prognosis [121]. Considering RSPOs in ovarian cancer, *in silico* analysis suggested relatively high *RSPO1* mRNA expression in ovarian cancer, and another study reported high expression of *RSPO1* and *RSPO3* in ovarian tumor xenograft material [116, 122]. Also, SNPs in the *RSPO1* locus have been identified as risk factors for ovarian cancers of serous histology [122, 123].

Moreover, a mouse study demonstrated that continuation of *Rspo1* expression after birth, normally downregulated in the ovaries at this stage, resulted in impaired ovulation and Wnt/β-catenin-mediated formation of granulosa cell tumors at the onset of puberty, suggesting that aberrant RSPO1 holds oncogenic potential in the ovaries [124].

#### Prostate

Wnt/β-catenin signaling is crucial during prostate development and both Wnt and RSPO ligands are expressed within the developing urogenital tract [125–128]. In vitro studies have indicated that RSPOs promote the growth and luminal differentiation in prostate organoid cultures [127, 129]. In prostate cancer, aberrant regulation of RSPOs and Wnt/β-catenin pathway components have been described [130–133]. *APC* and *CTNNB1* mutations are regularly found in prostate cancer [131, 133]. Moreover, *RSPO2* gene fusions associated with elevated *RSPO2* expression have been identified in prostate cancer patients, that were mutually exclusive with *APC* and *CTNNB1* mutations [131]. Unlike in CRC, these prostate cancer cases harbored fusions of *RSPO2* with *GRHL2* instead of *EIF3E* (ref. [131]). Also, *RSPO3* has been described as one of the genes being upregulated in prostate tumor stroma compared to healthy stroma [130]. In contrast, another group that studied gene expression data sets reported reduced levels of *RSPO3* in prostate tumors compared to healthy tissue, with further expression loss in metastatic disease and *RSPO3* loss correlating with an increased risk of relapse [132]. Thus, although *RSPO* fusions have been identified in prostate cancer patients and several reports have implicated RSPOs in prostate carcinogenesis, some controversy exists on the contribution of RSPOs to prostate cancer development.

### Other organs

In addition to aforementioned cancers, RSPO activation has been implicated in tumorigenic processes in more tissues. In the liver, the RSPO-LGR pathway has been defined as a key regulator of zonation, size and regeneration [134, 135]. Several reports have described *RSPO2* activation in liver cancer through distinct means [136–139]. Among these, *RSPO2* gene fusions have been identified, co-occurring with increased *RSPO2* expression levels, nuclear β-catenin localization and upregulation of Wnt target genes, resembling the situation of CRC cases with *RSPO2* gene fusions [137]. Several other studies reported subsets of hepatocellular carcinoma that harbor *RSPO2* copy number amplifications or enhanced *RSPO2* mRNA expression associated with Wnt/β-catenin activation [136, 138, 139]. Also, it has been shown that overexpression of *Rspo2* in a *Trp53* loss background caused tumor formation in the mouse liver [138]. In lung cancer, a protumorigenic role for RSPOs has been proposed as enhanced expression of RSPO ligands was observed in a subset of lung cancer cases, and enhanced *RSPO3* expression was associated with reduced patient survival [116, 140]. These studies reported no underlying *RSPO* gene fusions, and it was proposed that enhanced *RSPO3* expression might have resulted from promoter demethylation and deficiency in tumor suppressor *KEAP1* (ref. [116, 140]). Complementary in vitro and in vivo experiments suggested that RSPO3 promotes lung carcinogenesis through LGR4-IQGAP1 signaling [140]. Another group however did report *EIF3E-RSPO2* and *PTPRK-RSPO3* gene fusions in 1% and 2% of lung cancer patients respectively, being restricted to the squamous subtype of NSCLC [141]. Furthermore, enhanced *RSPO* expression and a tumor promoting role have also been described in pancreatic cancer and bladder cancer [116, 142, 143].

### Therapeutic targeting of RSPO in cancer

Hyperactivation of the Wnt/β-catenin pathway has been linked to tumor development in multiple organs, and the underlying molecular alterations are divergent. In line, compelling efforts have been made to develop therapeutic agents that target the Wnt/β-catenin pathway at various levels, among which those intervening with FZD or LRP receptor activity or Wnt ligand maturation and secretion through porcupine inhibitors (PORCNi) [144]. Also the RSPO receptor LGR5 is subject of investigation as a candidate target for therapeutic intervention in cancer [145]. With regard to Wnt driven cancers, dichotomous distinction can be made between ligand-independent and ligand-dependent cases, including those with *APC* or *CTNNB1* mutations versus those with *RSPO* or *RNF43* mutations respectively [55].

**Fig. 2:**
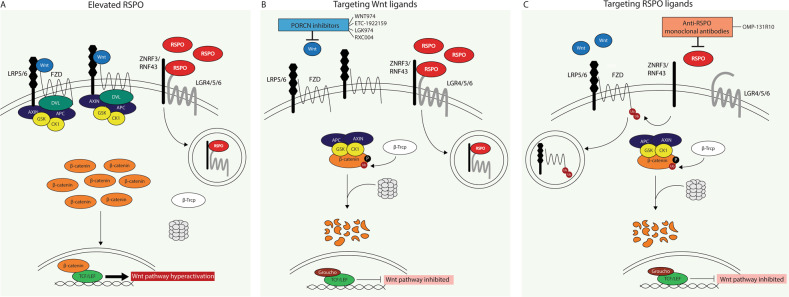
Schematic representation of therapeutic targeting opportunities in cancer cases with *RSPO* overactivation. **A** Overexpression of RSPOs induces increased clearing of negative regulators ZNRF3/RNF43 from the membrane, thereby expanding membranous Wnt receptor availability and excessive activation of Wnt ligand-mediated pathway activation. **B** PORCN inhibitors block the availability of functional Wnt ligands, allowing the destruction complex to form and degrade β-catenin, resulting in inhibition of the Wnt pathway. Indicated PORCNi are tested in clinical trials for solid cancers considering the RSPO status. **C** Monoclonal anti-RSPO antibodies disable RSPOs to clear negative regulators ZNRF3/RNF43 from the membrane, causing ubiquitination and membrane clearance of Wnt receptors, as such inhibiting Wnt ligands to activate the pathway. Indicated antibody has been tested in a clinical trial for colorectal cancer.

The ligand-dependent cases hold relatively more opportunities for targeted intervention. Specifically, with the growing indications for *RSPO* gene fusions/upregulation and a concomitant oncogenic role in several cancer types, RSPOs have emerged as promising candidate targets for therapeutic intervention and inherently as potential biomarkers predicting therapy responsiveness. Accordingly, some first studies have been published exploring the possibilities to inhibit tumor growth through targeting RSPO activity. These intervention strategies either directly targeted the RSPO ligands themselves or rather interfered more indirectly with Wnt ligand activity through PORCNi (Fig. 2). As the best described molecular activity of RSPOs lies within their capability. To amplify the signal of canonical Wnt ligands, aberrant RSPO expression would expectedly sensitize tumors to Wnt ligand blockade using PORCNi. Hence, PORCNi block the secretion of functionally active Wnt ligands and in their absence, RSPO ligands are impaired to exert any potentiating effects (Fig. 2B). Several preclinical studies have tested this using PORCNi in cancer cases with RSPO activation specifically [67, 146, 147]. Indeed, it was found consistently that PORCNi effectively reduced tumor growth whilst inducing tumor differentiation in PDX models of *RSPO*-fusion positive CRC and gastric cancers [67, 146, 147]. Currently, several clinical trials distinctively stratify cancer patients with genetic alterations in *RSPO2/3* as inclusion criteria to investigate the efficacy of PORCNi, either or not combined with other drugs [148–151]. As these trials specifically take into consideration the RSPO status of the considerate cancer patients, the drug efficacy data to be obtained by these trials will expectedly provide useful information for further decision making towards targeted intervention strategies in cancer patients with RSPO overactivation.

Direct targeting of RSPO proteins themselves with anti-RSPO antibodies represents another possible intervention approach (Fig. 2C). Through this means, RSPO ligands are disabled to clear ZNRF3/RNF43 from the membrane, leading to Wnt receptor degradation and thereby to inhibited Wnt pathway activation. Of additive value, direct targeting of RSPOs might also interfere with potential oncogenic signaling activities beyond stimulating canonical Wnt signaling. Hence, RSPOs have been implicated in other signaling pathways, though possible oncogenic roles there are insufficiently clear yet. In cancer cases with RSPO overactivation, direct targeting of RSPOs themselves might be favorable, and several studies have addressed the efficacy of anti-RSPO antibodies [58, 116, 152]. Chartier et al. generated monoclonal antibodies against RSPO1–3, and showed that these inhibited tumor growth (both as single agent or in combination with chemotherapy) in multiple PDX cancer models with overexpression of the respective *RSPO* [116]. These included an ovarian tumor with *RSPO1*, pancreatic and colon tumors with *RSPO2*, and lung and CRC tumors with *RSPO3* overexpression. Despite efficacy in most of the models, a minority of *RSPO* expressing tumors were not responsive [116]. Another study by Storm et al. used a CRC xenograft model specifically with a *PTPRK*-*RSPO3* gene fusion, and showed that anti-RSPO3 effectively reduced tumor growth and induced differentiation [58]. Both studies demonstrated that differentiation induced by anti-RSPO3 treatment was accompanied by downregulation of Wnt target and stem cell related genes [58, 116]. In addition, another study by Fisher et al. tested anti-RSPO3 treatment on CRC PDX models harboring *APC* mutations. Although these were not sensitive to anti-RSPO3 treatment only, the combination of anti-RSPO3 with paclitaxel synergistically reduced tumor growth in most cases, being accompanied by reduced nuclear β-catenin, proliferation and CSC frequency against enhanced differentiation [152]. In addition to these results within solid tumor models, a recent study also showed beneficial effects of anti-RSPO3 treatment in certain acute myeloid leukemia PDX models, where anti-RSPO3 treatment effectively inhibited leukemia stem cells without harming healthy stem cells [153].

Recognizing the promising clinical potential of RSPOs as novel therapeutic targets, a clinical trial has been set-up that tested the safety and efficacy of the neutralizing monoclonal anti-RSPO3 antibody OMP131-R10 (Rosmantuzumab) in cancer patients with advanced solid tumors and metastatic CRC [154]. It was reported that OMP131-R10 was well-tolerated by patients, though serum bone markers appeared affected [155]. The trial was unfortunately halted in phase I as a consequence of insufficient evidence for clinical benefit [155]. However it seems that the inclusion criteria for this trial did not take into consideration the RSPO status. In that case, it is unknown whether any and how many patients were included in the trial that actually had a gain in *RSPO3* specifically. Therefore, and given the multitude of indications for the relevant oncogenic role of RSPOs, it remains valuable to further investigate the clinical potential of anti-RSPO monoclonal antibodies specifically in cancer patients that harbor *RSPO* alterations.

Taken together, in line with the growing indications for the clinically relevant oncogenic role of RSPOs, some first avenues have been instigated to explore how we can potentially interfere with RSPO overactivation in cancer. Clinical trials addressing the efficacy of indirect and direct RSPO targeting strategies through PORCNi and anti-RSPO3 antibodies respectively will hopefully provide more insight beneficial to the development of novel treatment strategies against RSPO driven cancer.

## Conclusions and perspectives

RSPO ligands are powerful regulators of stem cell maintenance and tissue homeostasis. In accordance with this influential role, aberrant RSPO activation has increasingly been implicated in cancer development over the last decade. *RSPO* alterations, mostly represented by gene fusions or upregulation, have been reported to occur in patients of multiple cancer types. In addition, several studies have demonstrated that RSPO overactivation causally drives tumorigenesis in the mouse intestine, and provided indications that abnormal expansion of the stem cell compartment seems part of the mechanism. Most of our current knowledge on the molecular activities of RSPOs have been obtained by studies in the intestinal tract. Although these provide solid indications and relevant insight, only the first part of the puzzle seems uncovered, leaving many questions still unanswered. Among these, it remains unclear how the pathologic *RSPO* alterations are mechanistically achieved. Though specific breakpoints in the *RSPO* genes as well as specific fusion partner genes are involved in reported *RSPO* rearrangements, it is currently unknown how and under which conditions the *RSPO* fusions arise and are selected for along the tumorigenic cascade. A possible cell of origin for RSPO-driven cancer has not been reported yet, and its identification might be complicated by the fact that RSPOs are secreted proteins. Also, it is insufficiently clear what the specific molecular activities are that RSPO proteins instigate on receiving cells and that underpin carcinogenesis. Notably, for all these questions, the answers likely differ per organ.

Forthcoming, a better understanding on the molecular mechanisms of RSPOs with tissue-specific consideration is needed to provide well-founded directions for (pre)clinical studies. Current extensive indications for the oncogenic role of RSPOs however have already instigated the exploration of potential therapeutic opportunities and RSPOs have been recognized as promising therapeutic targets. Preclinical studies demonstrated that PORCNi and anti-RSPO antibodies efficiently inhibited tumor growth in PDX models of cancer with RSPO activation. Moreover, therapeutic targeting through both PORCNi and anti-RSPO3 antibodies are evaluated in clinical trials and will expectedly provide valuable information for further development of novel targeted intervention strategies.
